# Translation and Clinimetric Evaluation of SIPI, a Brief Instrument for Measuring the Complexity in Nursing Care

**DOI:** 10.1155/jonm/7258816

**Published:** 2026-09-03

**Authors:** José Manuel Romero-Sánchez, Elena Fernández-García, Olga Paloma-Castro, Juan Carlos Jiménez-Fernández, Davide Ausili, José Antonio Suffo-Aboza, Violeta Marín-Padillo, Ana Real-Tovar, Luis López-Molina

**Affiliations:** ^1^ Department of Nursing and Physiotherapy, University of Cádiz, Cádiz, Spain, uca.es; ^2^ Instituto de Investigación e Innovación Biomédica de Cádiz (INiBICA), Cádiz, Spain; ^3^ Department of Nursing, University of Seville, Seville, Spain, us.es; ^4^ Instituto de Biomedicina de Sevilla (IBiS), Seville, Spain; ^5^ Servicio Andaluz de Salud, Virgen Del Rocio University Hospital, Seville, Spain, huvr.es; ^6^ Department of Medicine and Surgery, University of Milano-Bicocca, Monza, Italy, unimib.it; ^7^ Department of Business Organization, University of Cádiz, Cádiz, Spain, uca.es

## Abstract

**Background:**

Measuring patient‐level complexity of nursing care in acute medical wards is essential for staffing and care planning, yet validated brief instruments in Spanish settings are scarce. The Sistema Informativo della Performance Infermieristica (SIPI) is a brief, intervention‐based index designed to quantify nursing‐care complexity on general wards.

**Aim:**

To translate SIPI into Spanish and evaluate its clinimetric properties in acute medical hospital wards.

**Methods:**

A cross‐sectional clinimetric instrument validation study. SIPI was translated into Spanish by two bilingual nurses, and a third bilingual nurse performed a back‐translation, which was reviewed by one of the original SIPI authors to ensure semantic equivalence. Construct validity was assessed in 212 hospitalised patients in four internal medicine wards. Convergent validity was examined using nurses’ perception of the complexity of care. Known‐groups validity was assessed by examining whether SIPI behaved as expected in relation to patient age and admission type. The optimal cut‐off was determined using a receiver operating characteristic analysis to evaluate discriminant validity.

**Results:**

The Spanish version was semantically equivalent to the original, with only minor wording adjustments. Convergent validity was supported: SIPI scores increased significantly across the four nurse‐rated complexity levels and were significantly higher in patients rated as high versus low complexity. In the ROC analysis, SIPI discriminated high‐ from low‐complexity patients with excellent accuracy (AUC = 0.94; 95% CI 0.91–0.97); the optimal cut‐off of 53.50 yielded 88.4% sensitivity, 86.0% specificity, 87.6% positive predictive value and 86.9% negative predictive value. Known‐groups validity was supported, as patients classified as high complexity by SIPI were significantly older than those classified as low complexity, and SIPI scores were significantly higher in urgent versus scheduled admissions.

**Implications for Nursing Management:**

The Spanish version of SIPI offers nurse managers a brief, patient‐level measure of nursing‐care complexity that could complement existing staffing and workload systems to support early identification of high‐complexity patients and more equitable, transparent resource allocation, pending confirmation in multicentre, outcome‐based studies.

**Conclusion:**

The Spanish version of SIPI showed adequate clinimetric properties, similar to the original Italian version, supporting its use as a brief instrument for measuring the patient‐level complexity of nursing care in acute medical hospital wards.

## 1. Introduction

The concept of complexity of nursing care has gained increasing relevance in healthcare systems as a critical determinant of both patient outcomes and resource allocation [1]. Complexity reflects the multifaceted needs of patients and the corresponding demands they place on nursing staff, which are not always accurately captured by medical diagnoses alone [2]. Despite its importance, measuring the complexity of nursing care in acute medical hospital wards remains challenging due to the limited availability of instruments validated for this specific purpose [1].

From a nursing science and nursing management perspective, the complexity of nursing care is a nursing‐sensitive construct that links patients’ care requirements to the professional resources needed to meet them safely; its accurate measurement is therefore crucial for evidence‐based staffing decisions and for ensuring patient safety and quality of care [2]. Longitudinal evidence supports an association between registered nurse staffing levels and patient outcomes, particularly in‐hospital mortality [3]. Patient classification systems and workload tools were developed precisely to translate patients’ care needs into staffing decisions; however, conventional approaches based on diagnosis‐related groups (DRGs) and volume‐based staffing categories have been criticised for failing to capture the actual nursing workload [4] and do not adequately account for patient‐level nursing‐care complexity [5]. This mismatch reinforces the need for instruments that quantify complexity directly from nursing interventions rather than inferring it from medical or administrative proxies.

Measuring the complexity of nursing care has followed different paths depending on the level at which decisions are made and the facet of complexity emphasised. At the ward level, acuity/dependency systems aim to balance capacity with demand. The RAFAELA system combines a daily patient classification with nurses’ professional judgement of optimal workload to set ward‐specific staffing targets [6]. The Safer Nursing Care Tool (SNCT) plays a similar role, offering empirically derived multipliers to estimate nurse staffing needs across medical‐surgical wards [7]. Nursing Hours Per Patient Day (NHPPD/HPPD) frameworks operationalise hours‐based staffing and have been linked to improvements in several nursing‐sensitive outcomes after implementation [8]. These approaches are valuable for capacity planning at the unit level.

In intensive care units, the dominant lens is time‐based workload measurement. The nursing activities score (NAS) attributes time weights to ICU activities to estimate required nursing time. Its predecessor, the Therapeutic Intervention Scoring System‐28 (TISS‐28), similarly quantifies intervention burden [9, 10]. These metrics excel at capturing work minutes in ICUs, but they do not directly represent the breadth and concurrency of fundamental nursing actions typical of general wards.

The Sistema Informativo della Performance Infermieristica (SIPI) [4] addresses this niche by offering a patient‐level, nursing intervention‐based measure of complexity tailored to general wards. SIPI is a brief grid‐based instrument comprising 18 binary items that document the presence or absence of specific nursing interventions performed in the previous 24 h. These items cover eight domains of patient care needs based on Cantarelli’s nursing model: breathing, feeding/hydration, elimination, hygiene, mobility, cardiac monitoring, diagnostic procedures and therapeutic procedures [11]. SIPI is modelled as a formative, clinician‐rated index, with items representing nursing‐care demand and serving as causal indicators of complexity [4]. The sum of positive responses generates a score ranging from 0 to 100, with higher scores indicating greater complexity of nursing care. In its original validation study, SIPI demonstrated substantial inter‐rater agreement and discriminative ability in classifying patients into low‐ and high‐complexity categories [4]. Moreover, it has been shown to predict in‐hospital mortality better than comorbidity indices [2], further underscoring its clinical utility. SIPI could complement ward‐calibration systems with a concise, documentation‐anchored view of patient‐level complexity on general wards.

In Spain, nurse staffing on hospital wards is still largely determined by fixed nurse‐to‐bed ratios and bed‐occupancy figures, which do not capture differences in patient‐level care complexity. Although Spanish initiatives based on the Nursing Interventions Classification have recently been proposed to measure nursing workload in adult hospitalisation units [12], validated instruments that quantify the complexity of nursing care for the individual patient remain scarce. For nursing managers, such an instrument would support more equitable workload distribution, the early identification of patients requiring intensified surveillance and more transparent, evidence‐informed staffing and resource‐allocation decisions at both clinical and managerial levels. SIPI is one such instrument, a brief, patient‐level, intervention‐based measure of nursing‐care complexity, although it had not been adapted or validated for use in Spain. This study addresses this gap by adapting SIPI into Spanish and evaluating its clinimetric properties in the Spanish hospital setting.

## 2. Methods

A two‐phase study was conducted. In the first phase, SIPI was adapted into Spanish, and in the second phase, its clinimetric properties were evaluated in a hospital sample.

### 2.1. Phase 1: Translation of SIPI Into Spanish

The translation and cross‐cultural adaptation of SIPI was informed by the core stages described in internationally recognised guidelines for adapting health measurement instruments [13, 14] and was adapted to the clinician‐completed, intervention‐based nature of SIPI. This procedure therefore represents a pragmatic adaptation of these established recommendations rather than their full application, tailored to the technical, clinician‐completed nature of SIPI and to its intended use. SIPI is not a patient‐reported outcome measure but a clinician‐completed, intervention‐based index: It is scored by nurses from the information documented in the patient’s clinical record, and its items denote standardised technical nursing interventions rather than patients’ subjective experiences or attitudes. Accordingly, the adaptation focused on achieving semantic, conceptual and operational, rather than experiential, equivalence of the items.

SIPI, originally developed in Italian, was independently forward‐translated into Spanish by two bilingual Italian–Spanish nurses whose native language was Spanish. Both translations were compared item by item to assess consistency, and discrepancies were reconciled through discussion until a consensus Spanish version was reached. A third bilingual nurse, whose native language was Italian and who was blind to the original instrument, back‐translated the consensus version into Italian. The back‐translation was then reviewed against the source version by one of the researchers who had participated in the original SIPI validation study to detect any semantic or conceptual discrepancy; where needed, wording was adjusted to ensure full equivalence with the original. Given the close linguistic kinship between Italian and Spanish and the technical, standardised nature of the items, this expert review served as the reconciliation and content‐verification step of the adaptation.

### 2.2. Phase 2: Evaluation of the Clinimetric Properties of SIPI

#### 2.2.1. Design

A cross‐sectional clinimetric instrument validation study was carried out. The study was conducted and reported in accordance with the STROBE guideline for cross‐sectional observational studies [15].

#### 2.2.2. Study Setting and Sampling

Participants were patients aged 18 years or older admitted to the four internal medicine wards of a regional hospital in Andalusia, southern Spain, between May and September 2023. Patients were required to be in an adequate clinical condition to provide consent and participate in the study within the first 24 h after admission. A consecutive sampling method [16] was used. Thus, all eligible patients assigned to the nurses collaborating in the project during the data collection period were invited to participate.

Because SIPI is a formative index (see Section 2.2.5), sample size adequacy was considered in relation to the planned clinimetric analyses, particularly hypothesis testing across theoretically relevant groups and receiver operating characteristic (ROC)–based estimation of discriminant accuracy. The final sample comprised 212 patients, with a balanced distribution after dichotomising nurses’ perceived complexity into low‐ and high‐complexity groups, which supported stable estimation of the ROC curve and its confidence interval (CI). The sample was therefore considered adequate for the validation aims of this single‐centre study, while acknowledging that larger multicentre samples are needed for external validation and cut‐off calibration.

#### 2.2.3. Variables and Instruments

The following variables and instruments were collected. Quantitative variables are followed by their unit of measurement and categorical variables by their possible values: sociodemographic—age (years) and gender (male, female, other); admission‐related data—reason for admission (scheduled, urgent due to the exacerbation of a chronic disease, urgent due to an emerging condition or transfers from another facility); and perceived complexity of care—the level of complexity perceived by the assigned nurse, measured on a four‐level scale (very low, medium–low, medium–high and very high). SIPI [4, 11]: The characteristics of SIPI were described in the introduction. The Spanish version of SIPI, developed in Phase 1 of this study, was used and is presented in the Supporting Table 1. A Spanish–English item mapping is provided in Supporting Table 2, using the English version of SIPI provided by Galimberti et al. [4].

#### 2.2.4. Data Collection

Fifteen collaborating nurses completed SIPI items within the first 24 h of the patient’s admission, based on the information recorded in the patient’s medical record. Before data collection, the 15 collaborating nurses attended a standardised two‐hour training session led by the research team. The session covered the operational definition and scoring rules of each of the 18 SIPI items and the specific clinical conditions in which each item applies and worked through practical cases using fictitious patients in which participants scored SIPI independently and then compared and discussed their ratings to calibrate interpretation and resolve doubts; it concluded with a debriefing. Each nurse also received the translated SIPI user guide (developed by the authors of the original version; Refs. [4, 11]), and a printed manual was available at every participating nursing station. Although these measures were intended to standardise scoring, formal post‐training agreement was not quantified (see Section 4.2). The nurses also collected sociodemographic data and admission‐related information from clinical records and recorded their perception of each patient’s care complexity using a four‐level scale. A custom‐built online survey platform was used to enter data, accessible via a password‐protected web link. Patient recruitment proceeded as follows: The collaborating nurses invited all eligible patients to participate in the study. Each nurse explained the study clearly, provided a written information letter and an informed consent form for the patient to sign and ensured that the patient’s signature was obtained. Since data were collected solely from the clinical records, the patient’s involvement concluded at that point.

#### 2.2.5. Statistical Analysis

Normality of the variables was assessed using the Kolmogorov–Smirnov–Lilliefors test [17]. Descriptive statistics were used to summarise quantitative variables using means and standard deviations and, for non‐normally distributed variables, medians, interquartile ranges, percentiles and the minimum and maximum; the shape of the SIPI score distribution was additionally described using skewness, and categorical variables were described using frequencies and percentages.

SIPI is conceived as a formative, causal‐indicator index: Its items are not interchangeable manifestations of a latent trait but distinct, complementary nursing interventions that together define, and cause, the construct of nursing‐care complexity [18, 19]. Three consequences follow for its validation. First, because formative indicators are not expected to covary, internal‐consistency coefficients such as Cronbach’s alpha are conceptually inappropriate, and a low inter‐item correlation is not evidence of poor measurement [20]. Second, factor‐analytic procedures and item response theory are not applicable, as they presuppose a reflective model in which items are effects of a common latent variable and are free to covary; in SIPI, items within each care domain are mutually exclusive forced‐choice options, producing an ipsative structure that violates this assumption [18]. Third, the validity of a formative index is therefore established not through its internal structure but through its relationships with external variables, that is, through criterion‐related and nomological construct validity [19]. This is precisely the strategy adopted here: convergent and known‐group validity tests whether SIPI behaves as theoretically expected against external referents, while the ROC analysis evaluates its criterion‐related discriminant accuracy against nurses’ judgement of complexity. These analyses interrogate the index’s external relationships rather than its internal dimensionality and are thus fully consistent with its formative nature.

Given the lack of an objective gold standard for the complexity of nursing care, convergent validity was assessed by comparing SIPI scores across the four levels of perceived complexity of nursing care. Because this reference reflects nurses’ expert clinical judgement rather than an objective external criterion, the convergent and discriminant results should be interpreted as agreement with expert appraisal rather than with an independent gold standard. Differences across levels were tested using the Kruskal–Wallis test, with post hoc pairwise Mann–Whitney *U* tests and Bonferroni‐adjusted *p* values. Evidence of adequate convergent validity was considered present when SIPI scores differed significantly across perceived‐complexity levels in the expected direction.

Discriminant validity was evaluated using ROC curve analysis to assess SIPI’s ability to correctly classify patients as having low or high complexity, with nurses’ perception as the reference standard. As the ROC curve does not allow for more than two levels, the ‘very low’ and ‘medium–low’ levels were combined into a single category of ‘low complexity of care’. In contrast, ‘medium–high’ and ‘very high’ were combined into ‘high complexity of care’. The area under the curve (AUC), standard error (SE) and 95% CI were calculated. An AUC between 0.80 and 0.90 was considered indicative of good accuracy, and an AUC above 0.90 was considered indicative of excellent accuracy [21]. SIPI score with the best sensitivity and specificity for distinguishing between low and high complexity was determined. Youden’s J statistic was used to identify the cut‐off point that maximised both sensitivity and specificity on the ROC curve. Youden’s J index is calculated as follows: sensitivity + (specificity – 1) and ranges from 0 to 1. In a perfect test, Youden’s index equals 1; values closer to 1 indicate greater discriminative ability [22]. In this study, specificity was defined as the probability that a patient perceived as low complexity by the nurse is also classified as low complexity by SIPI. At the same time, sensitivity referred to the probability that a patient perceived as high complexity by the nurse is also classified as high complexity by SIPI. The negative predictive value (NPV) was calculated as the probability that a patient classified as low complexity by SIPI is also perceived as such by the nurse, while the positive predictive value (PPV) is the probability that a patient classified as high complexity by SIPI is also perceived as such by the nurse.

Known‐group validity was assessed by examining whether SIPI behaved as expected in relation to patient characteristics theoretically associated with higher nursing‐care complexity [2]. First, age was compared between patients classified as low and high complexity according to the SIPI cut‐off. Second, SIPI scores were compared between scheduled and urgent admissions, under the assumption that urgent admissions would involve greater nursing‐care complexity. Both comparisons used the Mann–Whitney *U* test for independent samples.

Datasets containing missing values were excluded from analysis. A *p* value of < 0.05 was considered statistically significant. Data were analysed using IBM SPSS Statistics, Version 29.

#### 2.2.6. Ethical Considerations

The Ethics Committee of the Virgen Macarena and Virgen del Rocío University Hospitals (Seville, Spain) approved the study on 2 December 2022 (code 1578‐N‐22). Participation was voluntary for both nurses and patients. Patients were informed about the study and signed a written informed consent form. Anonymity and data confidentiality were guaranteed throughout the research process.

## 3. Results

### 3.1. The Spanish Version of SIPI

The direct translations into Spanish made by both independent translators were entirely equivalent. The back‐translation did not reveal concept‐level discrepancies. Only minor wording differences were identified and resolved. The Spanish version of SIPI is presented in Supporting Table 1, and Supporting Table 2 provides an item‐level mapping in English.

### 3.2. Sample Description

The sample was nearly balanced between men and women (50.9%, *n* = 108, vs. 48.6%, *n* = 103), with 0.5% identifying as transgender (*n* = 1). The mean age of the sample was 70.62 years (SD = 15.70). Most patients were admitted urgently due to either an emerging condition (54.2%; *n* = 115) or the exacerbation of a pre‐existing condition (33.5%; *n* = 71). Scheduled admissions and transfers from other facilities accounted for the remaining cases. Table 1 shows the demographic and clinical characteristics of the study sample (*N* = 212).

**TABLE 1:** Demographic and clinical characteristics of the study sample (*N* = 212).

Characteristic	Value
Age (years)	
Mean (SD)	70.62 (15.7)
Median (IQR)	73 (63–82)
Sex, *n* (%)	
Male	108 (50.9)
Female	103 (48.6)
Transgender	1 (0.5)
Reason for admission, *n* (%)	
Urgent—emerging condition	115 (54.2)
Urgent—exacerbation of a chronic disease	71 (33.5)
Scheduled	16 (7.5)
Transfer from another facility	10 (4.7)

*Note:* Percentages are calculated over the total sample (*N* = 212).

Abbreviations: IQR, interquartile range; SD, standard deviation.

### 3.3. Perceived Complexity of Care Distribution

In the four‐level classification, 36.8% (*n* = 78) were rated as medium–high, 33.5% (*n* = 71) as medium–low, 16% (*n* = 34) as very high and 13.7% (*n* = 29) as very low. When merged into a binary classification, 47.2% of patients (*n* = 112) were categorised as having high complexity and 52.8% (*n* = 100) were categorised as having low complexity, resulting in a balanced distribution.

### 3.4. SIPI Score Distribution

**FIGURE 1:**
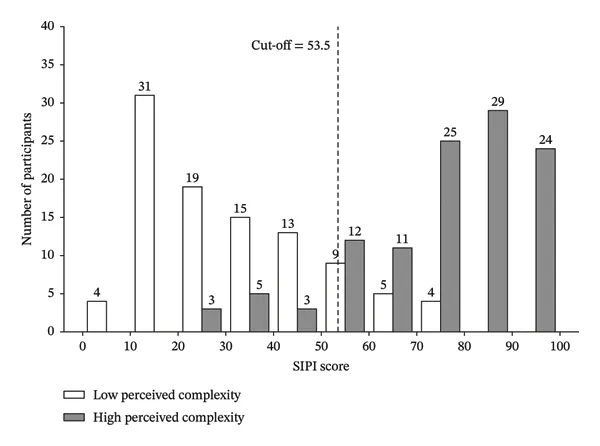
Distribution of participants across SIPI score ranges by perceived nursing complexity. Note: numbers in the bars represent the frequencies.

SIPI scores ranged from 0.00 to 98.30 (possible range 0–100), with a mean of 54.54 (SD = 27.88) and a median of 58.00 (25th–75th percentiles: 27.73–79.83; interquartile range = 52.10). The distribution departed from normality (Kolmogorov–Smirnov–Lilliefors *D* = 0.106, *p* < 0.001) but was approximately symmetric, with a slight negative skew (skewness = −0.16, SE = 0.17). The full distribution of scores across the perceived‐complexity groups is displayed in Figure 1.

### 3.5. Evidence of Construct Validity

#### 3.5.1. Convergent Validity

Statistically significant differences in SIPI scores were observed between patient groups classified according to nurses’ perceived complexity of care. These differences were evident in both the binary and four‐level classifications. As expected, higher perceived complexity was associated with higher SIPI scores. Significant differences were also detected in all post hoc pairwise comparisons among the four levels. Table 2 shows the descriptive statistics of SIPI scores for each level of perceived complexity, together with the results of the group‐comparison tests.

**TABLE 2:** Distribution of SIPI scores by the level of perceived complexity of care and group‐comparison results.

**Perceived complexity of care (2 levels)**					
	**Low**	**High**	**Group comparison**		

*n* (%)	100 (47.2)	112 (52.8)	*U* = 10520.5; *p* < 0.001		
SIPI score, M (SD)	32.0 (18.7)	74.7 (17.3)			

**Perceived complexity of care (4 levels)**					
	**Very low**	**Medium–low**	**Medium–high**	**Very high**	**Group comparison**

*n* (%)	29 (13.7)	71 (33.5)	78 (36.8)	34 (16.0)	*H* = 138.9; df = 3; *p* < 0.001^∗^
SIPI score, M (SD)	19.5 (11.3)	37.1 (18.7)	69.9 (17.4)	85.6 (11.3)	

*Note:* M, mean; *U*, Mann–Whitney *U*; *H*, Kruskal–Wallis. Percentages are calculated over the total sample (*N* = 212).

Abbreviations: df, degrees of freedom; SD, standard deviation.

^∗^Post hoc pairwise comparisons were performed using Mann–Whitney *U* tests with Bonferroni adjustment, and all six comparisons were statistically significant (*p* < 0.001).

#### 3.5.2. Discriminant Validity

**FIGURE 2:**
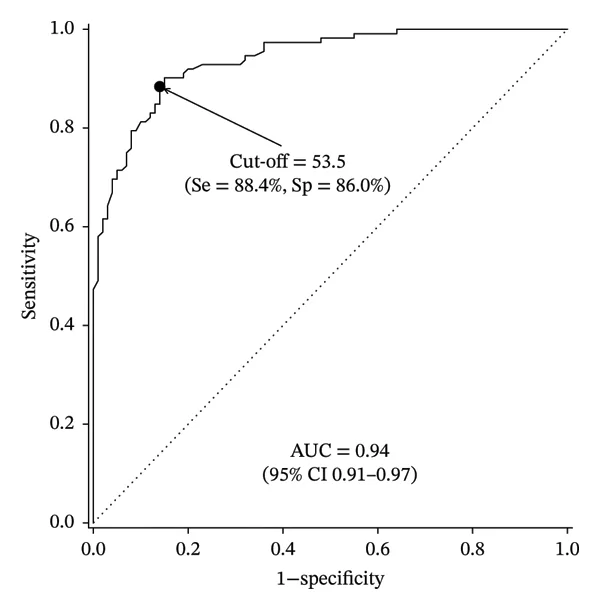
ROC curve with indication of the optimal cut‐off in two levels of nursing complexity (low and high) by SIPI score.

Figure 2 shows the ROC curve for SIPI scores classified into two levels of perceived complexity (low and high). The AUC was 0.94 (SE = 0.01; 95% CI = 0.91–0.97), significantly higher than the chance level (*p* < 0.001). The optimal cut‐off point was 53.50, yielding the highest Youden’s J value (*J* = 0.74), a sensitivity of 88.4% and a specificity of 86.0%. As shown in Figure 1, most patients perceived as having low complexity had SIPI scores below the cut‐off, whereas most patients perceived as having high complexity had scores above it. The PPV at this cut‐off was 87.6%, and the NPV was 86.9%. Misclassification was infrequent and broadly symmetric across groups (14 low‐complexity and 13 high‐complexity patients fell on the opposite side of the cut‐off; see Figure 1), indicating that the 53.50 threshold separated the two groups with comparable error in both directions.

#### 3.5.3. Known‐Groups Validity

Patients classified as high complexity by SIPI were significantly older, with a mean age of 75.56 years (SD = 12.40), compared to 65.28 years (SD = 17.19) in the low‐complexity group (*U* = 7368; *Z* = 4.41; *p* < 0.001). Likewise, patients with urgent admissions had significantly higher scores, with a mean of 55.47 (SD = 28.00), compared with 37.06 (SD = 20.23) in scheduled admissions (*U* = 899; *Z* = −2.46; *p* = 0.014).

## 4. Discussion

This study aimed to adapt SIPI into Spanish and to evaluate its clinimetric properties in a sample of hospitalised patients in Spain. SIPI items capture fundamental, evidence‐based nursing interventions that are largely standardised across European health systems, including Italy and Spain; therefore, substantial cross‐national cultural differences were not expected. The back‐translation did not reveal concept‐level discrepancies requiring modification, indicating that the Spanish version accurately reflects the original intended constructs. From a linguistic standpoint, only minor differences in wording were identified, given the close kinship between Italian and Spanish and the technical nature of SIPI items (e.g., ‘Monitorare la respirazione’ in Italian is translated as ‘Monitorizar la respiración’ in Spanish). These were harmonised by consensus, and, owing to the similarity between the two languages, the final Spanish wording is naturally very close to the original Italian, with no relevant changes in meaning.

The demographic and clinical profile of the sample was broadly comparable to those described in previous studies validating SIPI, with a similar distribution of sex and a predominance of older adults. The mean age in this study’s sample was around 70, slightly younger than the median age of 76 reported in a large Italian cohort [2], but still representing a high proportion of elderly patients typically admitted to internal medicine wards. As in the Italian studies, most patients were admitted urgently due to acute exacerbations of chronic disease or emergent conditions, highlighting the relevance of assessing complexity in populations with high acuity and comorbidities.

The mean SIPI score in the sample was approximately 54 points, slightly higher than the mean of almost 49 reported specifically for standard‐intensity care areas, such as internal medicine, in the Italian validation [4]. This modest difference is likely attributable to local differences in the patient casemix, such as higher levels of dependency or acuity.

The results supported evidence of SIPI’s convergent validity, as reflected in significant differences in scores across the four perceived‐complexity levels reported by nurses. Patients classified as having higher complexity showed consistently higher scores, with statistically significant pairwise differences between all levels. These findings align closely with those of the original Italian validation, in which SIPI scores increased progressively across perceived‐complexity levels, and significant discrimination between groups was observed [4]. These results from our sample underscore SIPI’s ability to reflect nurses’ expert judgement of practice complexity.

The ROC curve further supported the criterion‐related discriminative performance of SIPI. The AUC indicated excellent discriminative ability, slightly higher than the AUC reported in the Italian study [4]. The optimal cut‐off of around 53 points was identified, achieving high sensitivity (88.4%) and specificity (86.0%). The PPVs and NPVs were similarly high, indicating that SIPI effectively distinguishes between patients perceived as high‐ or low‐complexity by nurses. These findings are highly consistent with the original SIPI validation study, which proposed a cut‐off score of approximately 50, yielding classification accuracy comparable to that of the original validation study (sensitivity, 85%; specificity, 80%). The slightly higher cut‐off observed in our context is likely to reflect a combination of organisational and cultural differences between the Spanish and Italian settings rather than inconsistency of the index. In addition, the cut‐off in both validations was anchored to nurses’ subjective assessments of complexity. Local professional norms, workload expectations and cultural attitudes towards what constitutes a ‘complex’ patient may influence the optimal operating point. This pattern is consistent with other workload systems, such as RAFAELA [6], where thresholds are calibrated at the ward level rather than assumed to be universal. Taken together, the results suggest that SIPI showed adequate discriminative validity across healthcare systems. At the same time, its cut‐off values should be locally calibrated to cultural and organisational clinical realities to maximise interpretability and usefulness.

SIPI also evidenced known‐group validity, as patients classified as high complexity were significantly older than those classified as low complexity, and SIPI scores were significantly higher in urgent than in scheduled admissions. Older age [2] and admission urgency [2, 4] have previously been associated with increased complexity of nursing care and higher SIPI scores. The replication of these associations in the Spanish setting suggests that SIPI effectively captures key determinants of the complexity of nursing care for hospitalised patients.

### 4.1. Implications for Nursing Practice and Management

Given that registered nurse staffing levels are consistently associated with patient outcomes [3] and that the administrative indicators most often used to allocate nursing resources do not adequately account for patient‐level nursing‐care complexity [5], a brief, documentation‐anchored measure of complexity could be a useful complement to existing ward‐calibration and workload systems [6, 12]. At the point of care, SIPI’s ability to separate low‐ from high‐complexity patients may help charge nurses anticipate workload peaks, prioritise surveillance and technical procedures and recognise situations in which temporary adjustments to nurse‐to‐patient assignments or additional support could be considered, particularly when several very high‐scoring patients coincide on a ward. Aggregated over time, SIPI profiles could inform workload monitoring, more equitable patient allocation and local quality‐improvement initiatives. These potential applications should, however, be interpreted with caution: The present study establishes clinimetric validity in a single setting and does not test the effect of SIPI‐guided decisions on staffing, costs or patient outcomes. Whether SIPI can improve resource allocation or outcomes is an empirical question for prospective, ideally multicentre, intervention studies and the managerial uses outlined here remain provisional until such evidence is available.

### 4.2. Limitations

This study has several limitations that should be taken into consideration. First, the translation and cross‐cultural adaptation process did not include a formal multidisciplinary expert committee or pilot testing with nurse end users. Although the technical nature of SIPI items, the involvement of bilingual nurses and the review by one of the original SIPI researchers supported semantic, conceptual and operational equivalence, structured expert review and nurse end‐user pretesting would have strengthened the adaptation process. Future studies should therefore assess the clarity, feasibility and scoring consistency of the Spanish version among nurse end users before broader implementation.

Second, data were collected in a single regional hospital and were restricted to internal medicine wards, using a nonprobabilistic consecutive sampling strategy. Although consecutive sampling reduces some forms of selection bias by including all eligible patients within the recruitment period, the single‐centre design and the focus on a single type of ward limit the external validity of the findings. As discussed above in relation to the cut‐off, casemix, nurse‐to‐patient ratios and scope of practice may differ across hospitals, regions and clinical specialties, which constrains the transferability of the score distribution and the proposed cut‐off. The present results should therefore be regarded as context‐specific evidence of validity, and multicentre studies across diverse hospitals and ward types are needed before the Spanish version of SIPI can be generalised to the wider hospital population. SIPI itself was not initially developed for areas where nursing interventions differ substantially from those in internal medicine, such as paediatrics, obstetrics, psychiatry and intensive care.

Third, although SIPI showed excellent convergent validity against nurses’ expert judgements of complexity, this reference standard is inherently subjective. In the absence of a gold standard for patient‐level nursing‐care complexity, expert nurse judgement is nonetheless an accepted criterion in clinimetric validation; however, its subjectivity means the convergent‐validity findings should be read as supportive rather than definitive, and future studies should corroborate SIPI against more objective referents (e.g., measured nursing time, dependency indices or patient outcomes). In addition, the absence of well‐validated patient‐level complexity measures in Spanish precluded comparison with an established external instrument, limiting our ability to define a clear gold standard.

Fourth, the complexity of nursing care was assessed only once within the first 24 h after admission. Although this baseline SIPI score may still provide valuable information for early nursing resource allocation, it offers only a static snapshot of a construct that is inherently dynamic over the course of hospitalisation. Repeated assessments at later time points were not feasible with the available resources. Consequently, the cross‐sectional design, with a single SIPI measurement per patient, did not allow the evaluation of test–retest reliability or responsiveness to change. Longitudinal studies with repeated SIPI measurements at clinically meaningful time points are therefore needed to describe trajectories of nursing‐care complexity throughout hospitalisation and to confirm the index’s temporal stability and sensitivity to clinically important change.

Fifth, inter‐rater reliability of the Spanish version was not assessed. This was not feasible within the present design, as each patient was rated by a single nurse and paired independent ratings of the same patient were not collected. The standardised training and the translated scoring manual provided to all raters may have supported more consistent scoring, but they contextualise rather than resolve this limitation and do not replace formal reliability testing. Moreover, the inter‐rater reliability reported for SIPI items in preliminary studies of the original Italian version (88% agreement; Cohen’s *κ* = 0.78 [4]) cannot be assumed to transfer to the Spanish version or to other clinical contexts. Formal inter‐rater and intra‐rater reliability testing, together with post‐training competency checks, is therefore identified as a priority for future research before the Spanish SIPI is used at scale.

### 4.3. Future Research

Building on these findings, several avenues for future research are recommended. Future studies could adapt the SIPI for use in other specialised inpatient units and assess its performance in those contexts.

Additionally, SIPI accounts for only physiological nursing interventions, omitting important educational and psychosocial interventions, such as patient education and emotional support. These have been shown to improve key health outcomes in hospitalised patients, including treatment adherence and reduced readmissions [23–25]. Future research should explore integrating these dimensions into the SIPI framework to better reflect contemporary nursing practice.

Furthermore, the Spanish version of SIPI may serve as a basis for its adaptation to other Spanish‐speaking healthcare contexts. Future research should validate this approach in Latin America and other Spanish‐speaking regions, ensuring appropriate linguistic and cultural adjustments to reflect local nursing practices and care delivery models.

It would also be valuable for future studies to explore the predictive validity of SIPI scores for organisational and patient outcomes, such as workload distribution, length of stay and adverse events, to enhance its practical utility. Finally, studies could investigate how SIPI‐informed complexity profiles might be used to optimise staffing models and resource allocation.

## 5. Conclusion

The Spanish version of SIPI showed adequate clinimetric properties, comparable to those of the original Italian version. These findings support its use as a promising brief instrument for measuring the complexity of nursing care in Spanish hospitals. SIPI could complement ward‐calibration systems with a concise, documentation‐based view of patient‐level complexity on general wards, with the potential to support care planning and resource allocation, pending confirmatory, multicentre and outcome‐based research.

## Author Contributions

José Manuel Romero‐Sánchez: conceptualisation, methodology, formal analysis, writing–original draft, supervision and project administration. Elena Fernández‐García: methodology, investigation and writing–review and editing. Olga Paloma‐Castro: conceptualisation, methodology and writing–review and editing. Juan Carlos Jiménez‐Fernández: investigation, resources and data curation. Davide Ausili: methodology, validation and writing–review and editing. José Antonio Suffo‐Aboza: investigation, resources and data curation. Violeta Marín‐Padillo: investigation and data curation. Ana Real‐Tovar: investigation and data curation. Luis López‐Molina: methodology, formal analysis, visualisation and project administration.

## Funding

This research received no specific grant from any funding agency in the public, commercial or not‐for‐profit sectors.

## Conflicts of Interest

The authors declare no conflicts of interest.

## Supporting Information

Additional supporting information can be found online in the Supporting Information section.

## Supporting information


**Supporting Information** Supporting Table 1. Spanish version of the SIPI instrument (Panel A) and the scores assigned to each positive item (Panel B). Supporting Table 2. Spanish–English mapping of the SIPI items (Panel A) and the scores assigned to each positive item (Panel B).

## Data Availability

The data supporting the findings of this study are available from the corresponding author upon reasonable request.
