# Supplementary material for: Translation and Clinimetric Evaluation of SIPI, a Brief Instrument for Measuring the Complexity in Nursing Care
Source: J Nurs Manag. 2026 Sep 3;2026:7258816. doi: 10.1155/jonm/7258816 (PMC13540269; doi:10.1155/jonm/7258816)
Supplement: Supplementary file 1 [file JONM-2026-7258816-s001.docx]

**Supplementary Table 1:** Spanish version of SIPI (panel A) and scores assigned to each positive item (panel B).

| (A) PRINCIPALES ACTIVIDADES ENFERMERAS |  | |  | ***(B) PUNTUA-CIONES*** |
| --- | --- | --- | --- | --- |
|  | | |  |  |
| **1. ASEGURAR LA RESPIRACIÓN** | | |  |  |
| **1.a**. Mantener la permeabilidad de la vía aérea. | 🞏 SÍ | 🞏 no |  | *7.5* |
| **1.b**. Monitorizar la respiración. | **🞏 SÍ** | **🞏 no** |  | *2.5* |
|  | | |  |  |
| **2. ASEGURAR LA ALIMENTACIÓN Y LA HIDRATACIÓN** *– Únicamente puede marcarse sí en 1 ítem* | | |  |  |
| **2.a.** Facilitar la ingesta de alimentos y bebidas. | 🞏 SÍ | 🞏 no |  | *4.8* |
| **2.b**. Alimentar e hidratar por vía natural y/o artificial enteral. | **🞏 SÍ** | **🞏 no** |  | *12* |
|  | | |  |  |
| **3. ASEGURAR LA ELIMINACIÓN URINARIA Y FECAL** *- Únicamente puede marcarse sí en 1 ítem* | | |  |  |
| **3.a**. Favorecer la eliminación espontánea. | 🞏 SÍ | 🞏 no |  | *6* |
| **3.b.** Mantener el flujo descendiente de orina, heces y gases. | **🞏 SÍ** | **🞏 no** |  | *15* |
|  | | |  |  |
| **4. ASEGURAR LA HIGIENE** *- Únicamente puede marcarse sí en 1 ítem* | | |  |  |
| **4.a**. Ayudar parcialmente en la higiene y el vestido. | 🞏 SÍ | 🞏 no |  | *7.2* |
| **4.b.** Realizar la higiene al paciente. | **🞏 SÍ** | **🞏 no** |  | *18* |
|  | | |  |  |
| **5. ASEGURAR LA MOVILIDAD** *- Únicamente puede marcarse sí en 1 ítem* | | |  |  |
| **5.a**. Favorecer la marcha y el movimiento espontáneo. | 🞏 SÍ | 🞏 no |  | *3.6* |
| **5.b**. Proporcionar asistencia parcial para la marcha, la movilización y el posicionamiento. | **🞏 SÍ** | **🞏 no** |  | *10.8* |
| **5.c.** Proporcionar asistencia total para la marcha y el posicionamiento. | **🞏 SÍ** | **🞏 no** |  | *18* |
|  | | |  |  |
| **6. MONITORIZAR LA FUNCIÓN CARDIACA** | | |  |  |
| **6.a.** Monitorizar la función cardiaca. | **🞏 SÍ** | **🞏 no** |  | *8* |
|  | | |  |  |
| **7. LLEVAR A CABO PROCEDIMIENTOS DIAGNÓSTICOS** | | |  |  |
| **7.a.** Tomar muestras biológicas. | **🞏 SÍ** | **🞏 no** |  | *4.6* |
| **7.b**. Monitorizar el estado de consciencia. | **🞏 SÍ** | **🞏 no** |  | *2.4* |
|  | | |  |  |
| **8. APLICAR PROCEDIMIENTOS TERAPEÚTICOS** | | |  |  |
| **8.a.** Administrar tratamientos. | **🞏 SÍ** | **🞏 no** |  | *3.6* |
| **8.b.** Introducir y/o mantener la funcionalidad de sondas, catéteres y drenajes. | **🞏 SÍ** | **🞏 no** |  | *2.4* |
| **8.c.** Colocar o retirar dispositivos ortopédicos. | **🞏 SÍ** | **🞏 no** |  | *1.8* |
| **8.d.** Realizar vendajes y colocar apósitos. | **🞏 SÍ** | **🞏 no** |  | *4.2* |

**Supplementary Table 2:** Spanish–English mapping of SIPI items (panel A) and scores assigned to each positive item (panel B).

| (A) PRINCIPALES ACTIVIDADES ENFERMERAS  (*A) main nursing activities* |  | |  | ***(B) PUNTUA-CIONES***  ***SCORES*** |
| --- | --- | --- | --- | --- |
|  | | |  |  |
| **1. ASEGURAR LA RESPIRACIÓN**  ***1. Assure breathing*** | | |  |  |
| **1.a**. Mantener la permeabilidad de la vía aérea.  **1.a**. Maintain airway patency. | 🞏 Sí 🞏Yes | 🞏 no  🞏 no |  | *7.5* |
| **1.b**. Monitorizar la respiración.  **1.b**. Monitor breathing. | **🞏 Sí 🞏Yes** | 🞏 no  **🞏 no** |  | *2.5* |
|  | | |  |  |
| **2. ASEGURAR LA ALIMENTACIÓN Y LA HIDRATACIÓN** *– Únicamente puede marcarse sí en 1 ítem*  ***2. Assure feeding and hydration - only 1 item can be positive*** | | |  |  |
| **2.a.** Facilitar la ingesta de alimentos y bebidas.  2.a. Facilitate food and drinks intake. | 🞏 Sí 🞏Yes | 🞏 no  🞏 no |  | *4.8* |
| **2.b**. Alimentar e hidratar por vía natural y/o artificial enteral.  2.b. Feed and hydrate by natural and/or artificial enteral way. | **🞏 Sí 🞏Yes** | 🞏 no  **🞏 no** |  | *12* |
|  | | |  |  |
| **3. ASEGURAR LA ELIMINACIÓN URINARIA Y FECAL** *- Únicamente puede marcarse sí en 1 ítem*  ***3. Assure urination and defecation - only 1 item can be positive*** | | |  |  |
| **3.a**. Favorecer la eliminación espontánea.  3.a. Encourage the spontaneous elimination. | 🞏 Sí 🞏Yes | 🞏 no  🞏 no |  | *6* |
| **3.b.** Mantener el flujo descendiente de orina, heces y gases.  3.b. Maintain the down flow of urine, faeces and gas. | **🞏 Sí 🞏Yes** | 🞏 no  **🞏 no** |  | *15* |
|  | | |  |  |
| **4. ASEGURAR LA HIGIENE** *- Únicamente puede marcarse sí en 1 ítem*  ***4. Assure hygiene - only 1 item can be positive*** | | |  |  |
| **4.a**. Ayudar parcialmente en la higiene y el vestido.  4.a. Provide partial support for hygiene and clothing. | 🞏 Sí 🞏Yes | 🞏 no  🞏 no |  | *7.2* |
| **4.b.** Realizar la higiene al paciente.  4.b. Perform hygienic intervention. | **🞏 Sí 🞏Yes** | 🞏 no  **🞏 no** |  | *18* |
|  | | |  |  |
| **5. ASEGURAR LA MOVILIDAD** *- Únicamente puede marcarse sí en 1 ítem*  ***5. Assure Mobility - only 1 item can be positive*** | | |  |  |
| **5.a**. Favorecer la marcha y el movimiento espontáneo.  5.a. Encourage walking and spontaneous movement. | 🞏 Sí 🞏Yes | 🞏 no  🞏 no |  | *3.6* |
| **5.b**. Proporcionar asistencia parcial para la marcha, la movilización y el posicionamiento.  5.b. Provide partial assistance to walking, mobilization and placement. | **🞏 Sí 🞏Yes** | 🞏 no  **🞏 no** |  | *10.8* |
| **5.c.** Proporcionar asistencia total para la marcha y el posicionamiento.  5.c. Provide total assistance to walking and placement. | **🞏 Sí 🞏Yes** | 🞏 no  **🞏 no** |  | *18* |
|  | | |  |  |
| **6. MONITORIZAR LA FUNCIÓN CARDIACA**  ***6. Monitor Cardiac function*** | | |  |  |
| **6.a.** Monitorizar la función cardiaca.  6.a. Monitor cardiac function. | **🞏 Sí 🞏Yes** | 🞏 no  **🞏 no** |  | *8* |
|  | | |  |  |
| **7. LLEVAR A CABO PROCEDIMIENTOS DIAGNÓSTICOS**  ***7. Conduct diagnostic procedures*** | | |  |  |
| **7.a.** Tomar muestras biológicas.  7.a. Take biological samples. | **🞏 Sí 🞏Yes** | 🞏 no  **🞏 no** |  | *4.6* |
| **7.b**. Monitorizar el estado de consciencia.  7.b. Monitor the state of consciousness. | **🞏 Sí 🞏Yes** | 🞏 no  **🞏 no** |  | *2.4* |
|  | | |  |  |
| **8. APLICAR PROCEDIMIENTOS TERAPEÚTICOS**  ***8. Apply therapeutic procedures*** | | |  |  |
| **8.a.** Administrar tratamientos.  8.a. Administer treatments. | **🞏 Sí 🞏Yes** | 🞏 no  **🞏 no** |  | *3.6* |
| **8.b.** Introducir y/o mantener la funcionalidad de sondas, catéteres y drenajes.  8.b. Introduce and/or maintain the functionality of probes, catheters and drainage. | **🞏 Sí 🞏Yes** | 🞏 no  **🞏 no** |  | *2.4* |
| **8.c.** Colocar o retirar dispositivos ortopédicos.  8.c. Place or remove orthopaedic devices. | **🞏 Sí 🞏Yes** | 🞏 no  **🞏 no** |  | *1.8* |
| **8.d.** Realizar vendajes y colocar apósitos.  8.d. Bandaging and medical dressings. | **🞏 Sí 🞏Yes** | 🞏 no  **🞏 no** |  | *4.2* |
